# Capillary density has no value as an early biomarker of bevacizumab efficacy in metastatic colorectal cancers: a prospective clinical trial

**DOI:** 10.18632/oncotarget.22822

**Published:** 2017-12-01

**Authors:** Jean-David Fumet, Aurélie Bertaut, Leila Bengrine, Patricia Lapierre, Julie Vincent, François Ghiringhelli, Nicolas Falvo

**Affiliations:** ^1^ Department of Medical Oncology, Centre Georges-François Leclerc, Dijon, France; ^2^ Department of Epidemiology and Biostatistics, Georges François Leclerc Center, Dijon, France; ^3^ Center of Clinical Research, Georges François Leclerc Center, Dijon, France; ^4^ Department of Radiology, François-Mitterrand Teaching Hospital, Dijon, France

**Keywords:** angiogenesis, bevacizumab, capillary rarefaction, colorectal cancer

## Abstract

**Background:**

Bevacizumab is a recombinant humanized monoclonal immunoglobulin G1 antibody targeting VEGF-A. It is currently used with chemotherapy as the first- or second-line therapy in metastatic colorectal cancer. Previous studies have showed that anti-angiogenic agents decrease capillary density. We evaluated the link between decreased capillary density and the response to bevacizumab-based chemotherapy.

**Results:**

Overall, 43 patients with metastatic colorectal cancer treated with first-line bevacizumab-based chemotherapy were enrolled. At Day 90, progressive disease was observed in 12 patients (27.9%). All patients presented decreased capillary density. ROC analysis at different time points and capillary density variation showed a poor diagnostic performance regarding response at Day 90.

**Materials and Methods:**

From 2013 to 2015, patients with metastatic colorectal cancer treated in our French cancer care center and eligible for bevacizumab with chemotherapy were enrolled in a prospective single-center study. Capillary density was assessed using capillaroscopy at Day 1, Day 15 and Day 30. Response to bevacizumab was assessed at Day90 according to CHUN criteria.

**Conclusions:**

Capillary density measured using capillaroscopy is not a good predictor of the early response to bevacizumab-based chemotherapy. (NCT01810744).

## INTRODUCTION

Colorectal cancer (CRC) is the third most common cancer worldwide [1]. Approximately 25% of patients present with metastases at the initial diagnosis and almost 50% of patients with CRC will develop metastases [2]. Fluorouracil-based palliative chemotherapy has been the mainstay treatment for many years. The availability of the cytostatic drugs irinotecan and oxaliplatin has improved the prognosis of metastatic CRC (mCRC) patients. In the last decade, a new class of « anti-angiogenic » target therapies have been included in treatments for colorectal cancer and several other cancers (Breast cancer, renal clear cell carcinoma, ovarian cancer or non-small cell lung cancer) [3–8]. Neoangiogenesis results from an imbalance between pro-angiogenic factors like VEGFs (Vascular Endothelial Growth Factor) and anti-angiogenic factors. This mechanism is known to be required for tumor growth and the development of metastasis. VEGF-A, a diffusible glycoprotein produced by normal and neoplastic cells, is one of the most important regulators of physiological and pathological angiogenesis [9]. VEGF-A-dependent angiogenesis, which contributes to tumor cell proliferation, is thus a target for cancer therapy.

Several anti-angiogenic target therapies are currently used in the treatment of human cancers. Bevacizumab is a recombinant humanized monoclonal immunoglobulin G1 antibody targeting VEGF-A. It has been approved for the treatment of advanced colorectal, breast, lung, renal, and ovarian carcinomas [4–8, 10]. Adding bevacizumab to currently used chemotherapy improves outcomes in patients with mCRC. Indeed, Bevacizumab, when used in the first- and second-line treatment of mCRC, improved progression-free survival (PFS) and overall survival (OS) [7, 8, 11, 12]. In recent clinical trials, the median OS of patients with mCRC treated with chemotherapy alone was around 13 to 20 months but the median OS of patients with mCRC treated with bevacizumab combined with chemotherapy was more than 25 months [7, 8, 13]. However, to date no predictive or early biomarker of efficacy has been validated.

One of the most frequent side effects of treatment with bevacizumab is hypertension (HT). The perfusion of exogenous VEGF-A is accompanied by hypotension through the activation of endothelial NO-synthase and the production of nitric oxide [14, 15]. It was therefore suggested that the pathophysiology of hypertension induced by bevacizumab or other antiangiogenic agents may be due to neutralization of the major physiological effects of VEGF-A on endothelial cells and therefore on the vascular wall. This hypothesis implies that the inhibition of endothelial NO synthase in response to various stimuli may alter the endothelial vasculature and increase sympathetic adrenergic activity [14]. Together, such biological events should lead to capillary rarefaction. Capillaries are the smallest blood vessels in the human body with a function in the exhange of materials between blood and tissue. Capillary density is defnied as the count of perfused capillary per mm2. It has been used as an indicator of the quality of issue perfusion.

Capillaroscopy is a non-invasive, easy and safe diagnostic technique designed to evaluate small vessels of the microcirculation in the nailfold and thus evaluate capillary density. In the nailfold, terminal rows of capillaries run parallel to the skin surface and, therefore, all morphological details and the nature of the blood flow can be examined. Capillaroscopy allows the precise qualitative and quantitative evaluation of the microcirculation and is a valuable tool in the daily practice of rheumatologists [16].

To our knowledge, only two studies with 14 and 16 patients, respectively, have described a decrease in capillary density during treatment with anti-angiogenic therapy and observed a correlation between capillary density reduction and bevacizumab efficacy in various types of cancer [17, 18]. However, this observation has not been reproduced prospectively in a larger and more homogenous cohort of patients in the setting of a clinical trial.

We therefore investigated variations in capillary density in patients with mCRC treated with bevacizumab-based therapy and determined their relationship with the response to treatment, PFS and OS.

## RESULTS

### Patients’ characteristics

Forty-three patients with metastatic colon cancer were enrolled. The main demographic and clinical characteristics of the patients are listed in Table 1. Overall, 47.6% of patients had right-side colon cancer, 28.6% of patients had left-side colon cancer and 23.8% of patients had rectal cancer. Of these patients, 11 (25.6%) had Wild Type (WT) K-RAS status, 21 (48.9%) had K-RAS exon 2 mutated status and four (9.3%) had BRAF-mutated status. Seven (16.2%) patients had unknown status. Around half of the patients had surgery for their primary tumor, with R0 resection for the vast majority (93%). All included patients were in the first-line of metastatic treatment. All patients except one received bi- or tri-chemotherapy (Table 1).

**Table 1:** Baseline characteristics of the study population

			Total	
			*n* = 43	(%)
**Age, years**	mean (sd)	66.51 [31.91–90.25]		
**Sex**				
men			22	51,16
women			21	48,84
**Primary disease site**				
right-sided colon			20	47,62
left-sided colon			12	28,57
Rectum			10	23,81
Missing			1	
**KRAS**				
WT			11	28,95
mutated			27	71,05
Missing			7	
**BRAF**				
WT			30	88,24
Mutated			4	11,76
Missing			9	
**Surgery of primary site**				
no			21	48,84
yes			22	51,16
**Quality of surgery**				
R0			13	92,86
R1			1	7,14
Missing			8	
**pT (*n* = 22)**				
2			1	5,26
3			8	42,11
4			9	47,37
5			1	5,26
Missing			3	
**pN (*n* = 22)**				
0			6	30
1			7	35
2			6	30
X			1	5
Missing			2	
**pM (*n* = 22)**				
0			11	55
1			9	45
Missing			2	
**Adjuvant chemotherapy**				
no			32	74,42
yes			11	25,58
**Chemotherapy regimen associated with bevacizumab**				
Oxaliplatine + Irinotecan + Fluoropymidine			7	16,28
Oxaliplatine + Fluoropymidine			28	65,12
Fluoropymidine + Irinotecan			7	16,28
Fluoropymidine			1	2,33
**Metastasis**				
Synchronous			30	69,77
Metachronous			13	30,23
**Prior hypertension**				
no			30	69,77
yes			13	30,23

### Survival and response rates

**Figure 1:**
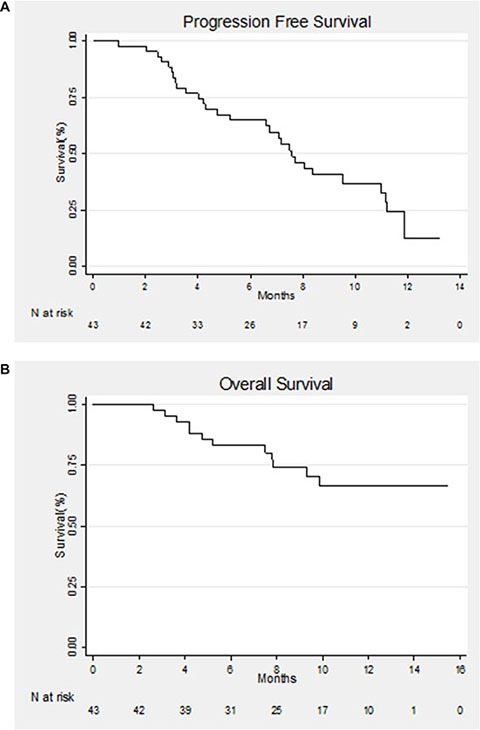
(**A**) Kaplan–Meier estimate of progression-free survival (PFS) for all patients. (**B**) Kaplan–Meier estimate of overall survival (OS) for all patients.

The median follow-up was 11.7 months (0.95–15.47). Overall, after 1 year of follow-up, 30 patients (69.7%) had progressive disease and 12 patients (27.9%) had died. Median PFS was 7.6 months (95% CI: 5.2–11]. PFS at 6 months was 65% (95% CI: [48.5%–77%]) while PFS at one year was 12% (95% CI: [2.5%–30%]) (Figure 1A).

OS at 6 and 12 months was 83% (95% CI: [68%–91.5%]) and 66% (95% CI: [47.5%–80%]), respectively (Figure 1B).

Three months after the initiation of therapy, 10 patients had progressive disease and two had died.

### Vascular assessment

**Figure 2:**
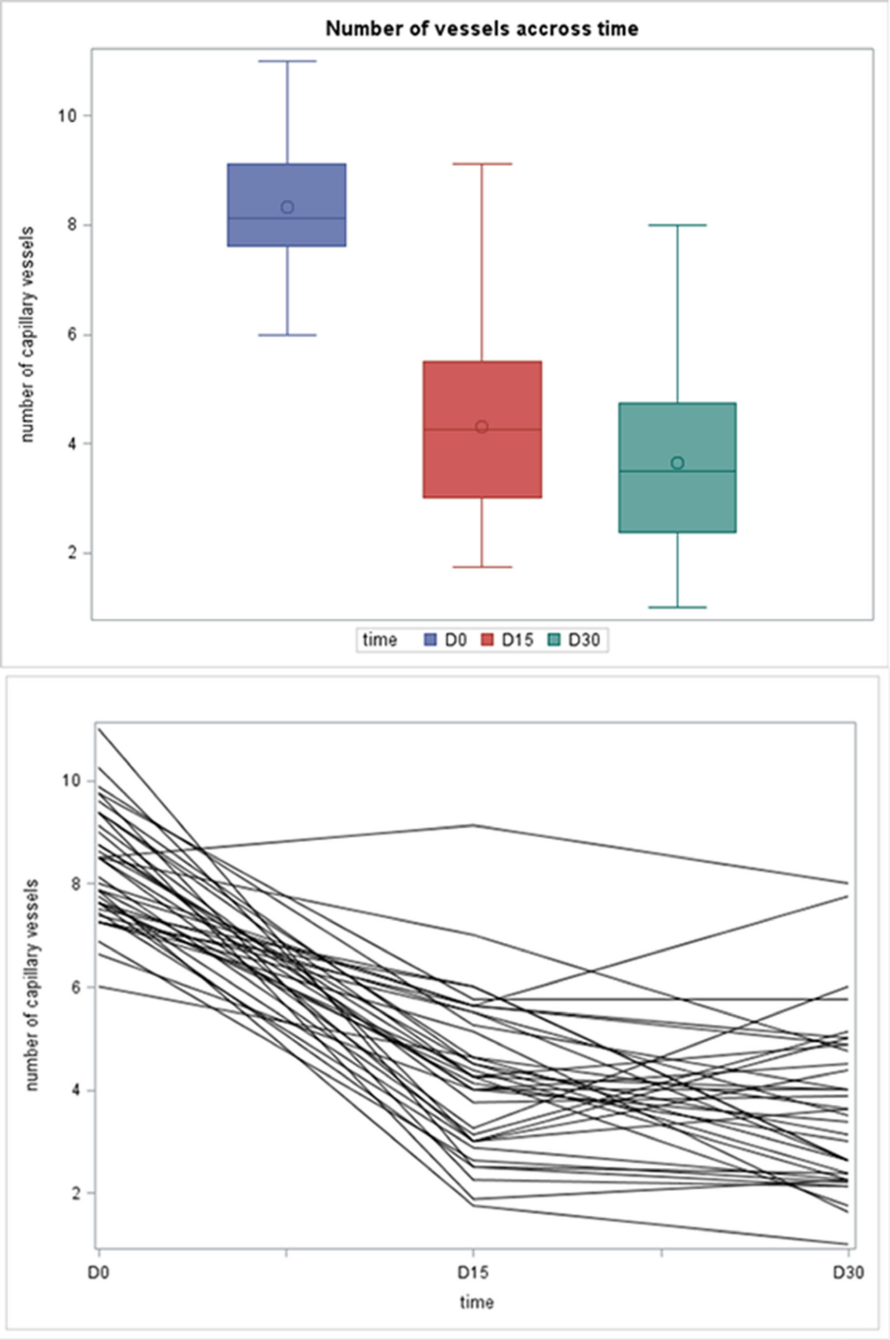
(**A**) Boxplot of capillary density at Day 0, Day 15 and Day 30. (**B**) Capillary density accross time.

Capillary density was measured in 37 patients during treatment with bevacizumab. For the six remaining patients, no valid capillaroscopy assessments of capillary density could be obtained because of technical problems. During the treatment with bevacizumab, capillary density had significantly decreased on days 15 and 30. The median capillary count was 8.19 [6.00–11.00] before therapy and 4.25 [1.75–9.13] and 3.50 [1.00–8.00] after 2 weeks and 4 weeks of therapy, respectively (Table 2). All of the patients except one showed a decrease in the capillary count at day 15 and all patients at day 30 (Figure 2A–2B).

**Table 2:** MAP and capillary density at 3 time point (Day 0, Day 15 and D 30)

	Day 0	Day 15	Day 30
MAP			
*n*	36	43	40
Mean (SD)	97.6 (11.1)	98.5 (10.8)	101.1 (11.3)
Median (min-max)	95.3 (81.7 – 132.3)	99.3 (73 – 124.3)	100.8 (79.7– 127)
Capillary density			
*n*	37	37	37
Mean (SD)	8.33 (1.10)	4.30 (1.53)	3.65 (1.61)
Median (min-max)	8.13 (6.00–11.00)	4.25 (1,75–9.13)	3.50 (1.00–8.00)

**Figure 3:**
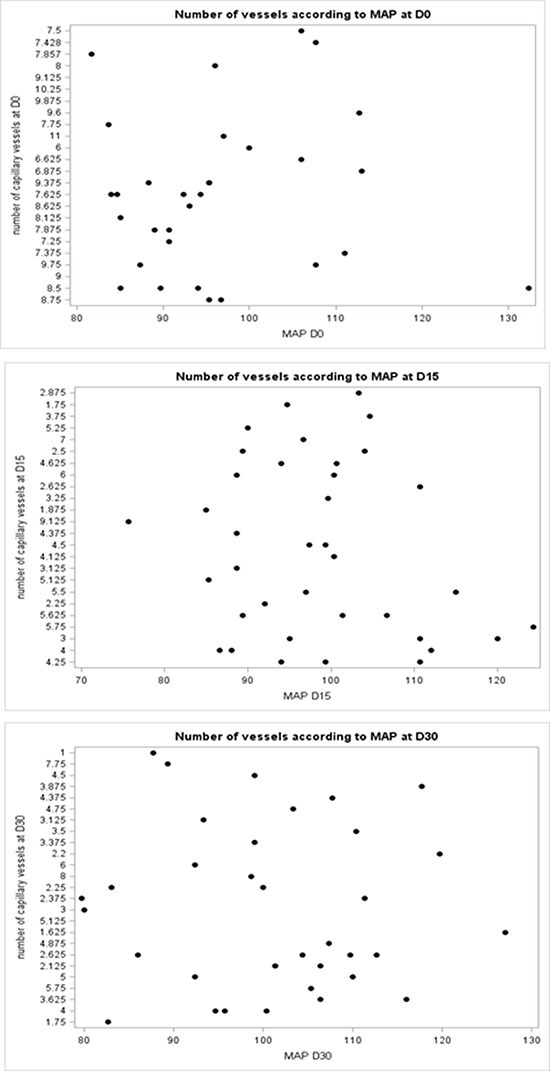
A scatter plot did not show correlation between capillary density and MAP at any time point (Day0, Day15 and Day30)

Mean arterial blood pressure (MAP= (TAS+2TAD)/3) was measured in all patients. MAP was 95.33 mmHg [81.66–132.33] before therapy and 99.33 mmHg [73–124.33] and 100.83 [79.66–127] after 2 weeks and 4 weeks of therapy, respectively (Table 2). MAP had increased in 50% and 51.2% of patients at day 15 and day 30, respectively. We found no correlation between capillary density and MAP at any time point (Figure 3).

**Figure 4:**
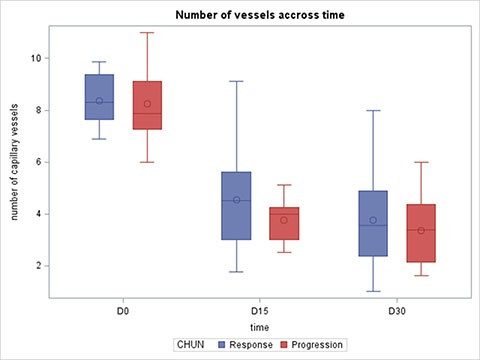
Capillary density at Day 0, Day 15 and Day 30 according to response to treatment

In both responders and non-responders, we found a decrease in capillary density at D15 and D30 after the initiation of therapy. There were no significant differences between these two groups in terms of capillary density at D1, D15 or D30 (Figure 4). Furthermore, no significant difference was found between the two groups for capillary density variation between D0 and D15, D0 and D30 and D15 and D30 (data not shown).

**Figure 5:**
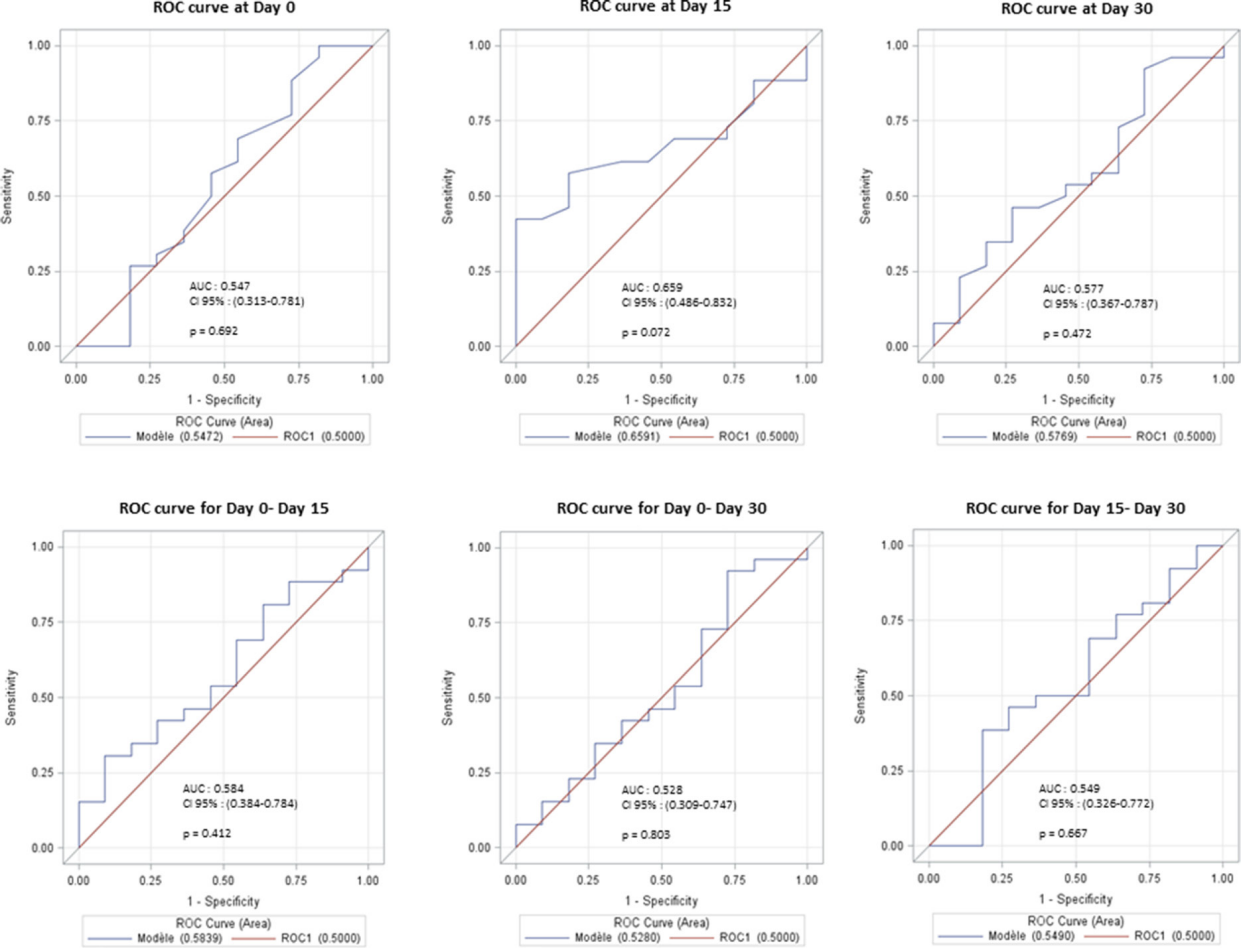
(**A**) ROC curves modelling the relationship between capillary density at Day 0, Day 15 and Day 30 on response at Day 90. (**B**) ROC curves modelling the relationship between capillary density variation between Day 0–Day 15, Day 0–Day 30 and Day 15–Day 30 on response at Day 90.

Regarding absolute capillary counts, the area under the ROC (Receiver Operating Characteristic) curve (AUCs) (Figure 5A) were 0.54 (*p* = 0.69; 95% CI [0.3134-0.7810]), 0.66 (*p* = 0.07; 95% CI [0.4858-0.8324] and 0.58 (*p* = 0.47; 95% CI [0.3673-0.7866]), at D0, D15 and D30, respectively. (Figure 5A).

Concerning relative variations in capillary density between D0 and D15, D0 and D30, D15 and D30, the AUCs were 0.58 (*p* = 0.41; 95% CI [0,38-0,78]), 0.52 (*p* = 0.80; 95% CI [0,31-0,75]) and 0.55 (*p* = 0,67; 95% CI [0,32–0,77]), respectively. (Figure 5B).

Similarly, none of these capillary density parameters could predict PFS or OS (data not shown).

## DISCUSSION

Bevacizumab is one of the most frequently used target therapies associated with chemotherapy for metastatic colorectal cancer. To our knowledge, there are no biomarkers to predict its efficacy.

In this study, based on prospectively recorded data from a clinical trial and capillary density assessed using capillaroscopy, we found a decrease in capillary density in patients treated with bevacizumab. These findings are in agreement with previous studies. Indeed, Mourad et al. [17] showed that VEGF-A inhibition by bevacizumab caused a decrease in capillary density. Another study found the same results and demonstrated the reversibility of this phenomenon when bevacizumab therapy was stopped [18, 20]. Interestingly, we observed a decrease in capillary density in all patients, thus demonstrating that bevacizumab had biological activity on normal nailfold microvasculature. Bevacizumab is used at 5 mg/kg every 2 weeks in colorectal cancer and at 10 mg/kg every 2 weeks in other diseases. It would be interesting to compare the effect of the two dosages on capillary density to determine whether increasing the dose increases the magnitude of the biological efficacy.

Previous publications reported that increased blood pressure during treatment with VEGF inhibitors has been associated with a longer time to tumor progression [21]. Surprisingly, we found no correlation between blood pressure and capillary density. Moreover, we found no association between a rapid increase in MAP and the response to therapy. We believe that these results show that increased MAP could not be considered a potential early biomarker of bevacizumab efficacy.

Very few studies have evaluated the correlation between variations in capillary density and survival. Capillaroscopy is a cheap, non-invasive method and capillary density could have been an interesting predictive marker of bevacizumab efficacy. Unfortunately, it would seem that capillary density is not useful to predict the efficacy of bevacizumab. Nailfold capillaries evaluated by capillaroscopy may not be a good surrogate of capillary density in the tumor. However, these negative results were also found in a study with a small number of patients, and the lack of any difference between responders and non-responders could be related to a lack of power.

It should nevertheless be noted that median PFS in our population was lower than that in phase 3 trials using bevacizumab in the first line (7.6 months in our cohort versus around 10 months in a recent trial) [7, 8]. Most recent studies demonstrated poorer survival in right-side compared with left-side colon cancer [24]. The high prevalence of right-side colon cancer in our population could explain this poor survival rate and may have affected our initial statistical hypothesis. Furthermore, our patients were not treated with the same bevacizumab-based chemotherapy. We could imagine that this heterogeneity may have altered the interpretation of our results.

In conclusion, in light of the findings of this prospective study, we can say that treatment with bevacizumab results in a rapid and persistent decrease in capillary density in all patients and that this decrease is not associated with a response to bevacizumab at Day 90.

## MATERIALS AND METHODS

### Patients

Patients with histologically confirmed metastatic colorectal cancer and eligible to receive first-line treatment with bevacizumab combined with chemotherapy were enrolled from May 2013 to July 2015 at the Georges Francois Leclerc Cancer Center in Dijon, France. Written informed consent was obtained for all patients before inclusion (NCT01810744). Bevacizumab was given at 5 mg/kg every 2 weeks or 7.5 mg/kg every 3 weeks depending on the chemotherapy regimen. Blood pressure was measured at each visit.

Tumor response was defined according to CHUN criteria [19].

### Study design

In all patients, after inclusion and prior to the initiation of treatment, baseline capillary density was evaluated using capillaroscopy and blood pressure was measured.

An evaluation with a medical consultation, capillaroscopy and blood pressure was carried out during three key visits (Day 15, Day 30 and Day 90).

Tumor response to chemotherapy was evaluated by CT-Scan on day 90 using CHUN criteria. From day 90, data on treatment response, PFS and OS were reported every 3 months until 1 year.

### Capillary assessment

Nail fold capillaries in the dorsal skin of the third finger were visualized by a capillary microscope. Capillary density was defined as the number of erythrocyte-perfused capillaries per mm². We used a video microscope system Cap-Xview HD (XPort Technologies).

Baseline capillary density corresponded to the number of functional capillaries. Each finger of each hand except the thumb was then observed one by one, around the entire circumference of the lunula. The capillary density was defined as the mean of the eight fingers.

The number of capillaries was counted off-line by an experienced investigator (Nicolas Falvo).

### Statistical analysis

Quantitative variables were described using means and standard deviations or medians and ranges. Relationships between variables and the CHUN response were analyzed using a non-parametric Wilcoxon test. Qualitative variables, excluding missing data, were described using percentages and compared using Chi² or Fisher tests. Median follow-up was calculated using the reverse Kaplan-Meier method. Survival probabilities were estimated using the Kaplan-Meier method, and the log-rank test was used to compare survival curves.

Relative variations in capillary density were calculated as follows: delta D0–D15 = (number of capillaries at D0-number at D15)/number at D0. The same formula was applied for delta D0–D30 and D15–D30. The prognostic value of absolute capillary density at D0 and deltas of capillary density were determined using areas under the ROC curves with their 95% confidence intervals (CI).

Statistical analyses were performed using SAS 9.4 software. All tests were two sided and *P* values were considered significant when less than 0.05.

## Abbreviations

CRC: Colorectal cancer
mCRC: metastatic Colorectal cancer
VEGF: Vascular Endothelial Growth Factor
PFS: progression-free survival
OS: overall survival
MAP: Mean arterial blood pressure
D1: day 1
D15: day 15
D30: day 30
ROC: Receiver Operating Characteristic
